# COMPASSIONATE CAREGIVING: ENHANCING GERIATRIC SUPPORT THROUGH GWEP CNA INITIATIVES

**DOI:** 10.1093/geroni/igae098.0896

**Published:** 2024-12-31

**Authors:** Samantha Cotton

**Affiliations:** Chair

## Abstract

The Geriatric Workforce Enhancement Programs (GWEPs) at various institutions have launched innovative educational efforts to support Certified Nursing Assistants (CNAs), a key yet often overlooked segment of the healthcare workforce. These initiatives strive to mitigate high turnover rates and fill educational gaps, with a special focus on leadership, geriatric care, and compassionate handling of individuals with Alzheimer’s disease and related dementias (ADRD). The University of Chicago Medicine’s SHARE Network introduced the CNA Leadership Academy, aimed at nurturing CNAs’ leadership capabilities through a curriculum that includes leadership principles, quality improvement, and resilience, facilitated by Project ECHO’s tele-mentoring. The University of California-Irvine GWEP’s program, “Building Resiliency While Advancing Your Caregiver Skills,” targeted geriatric care enhancements, incorporating the 4-Ms and strategies for challenging behaviors through interactive and trauma-informed teaching methods. Meanwhile, the University of Louisville’s GWEP developed a compassionate care model for CNAs in long-term care, offering training via online modules, workshops, and an iOS app to improve management of ADRD-related behaviors. These GWEPs collectively aim to bolster CNAs’ roles in healthcare, equipping them with essential skills to elevate patient care, especially in geriatric and dementia settings. By enhancing CNAs’ abilities and job satisfaction, these programs contribute to a more resilient, knowledgeable, and empathetic geriatric care workforce, demonstrating a comprehensive effort to advance the quality of care for older adults. This symposium will focus on the critical need of strengthening the training of CNAs and lessons learned across our programs.

